# Individual variation in whole-animal hypoxia tolerance is associated with cardiac hypoxia tolerance in a marine teleost

**DOI:** 10.1098/rsbl.2015.0708

**Published:** 2016-01

**Authors:** William Joyce, Karlina Ozolina, Florian Mauduit, Hélène Ollivier, Guy Claireaux, Holly A. Shiels

**Affiliations:** 1 Faculty of Life Sciences, University of Manchester, Manchester, UK; 2 Centre Ifremer de Brest, Université de Brest, LEMAR (UMR-6539), Unité PFOM-ARN, Plouzané, France

**Keywords:** individual variability, climate change, hypoxic dead zones, heart, sea bass

## Abstract

Hypoxia is a pervasive problem in coastal environments and is predicted to have enduring impacts on aquatic ecosystems. Intraspecific variation in hypoxia tolerance is well documented in fish; however, the factors underlying this variation remain unknown. Here, we investigate the role of the heart in individual hypoxia tolerance of the European sea bass (*Dicentrarchus labrax*). We found individual whole-animal hypoxia tolerance is a stable trait in sea bass for more than 18 months (duration of study). We next examined *in vitro* cardiac performance and found myocardial muscle from hypoxia-tolerant individuals generated greater force, with higher rates of contraction and relaxation, than hypoxic-sensitive individuals during hypoxic exposure. Thus, whole-animal hypoxia tolerance is associated with cardiac hypoxia tolerance. As the occurrence of aquatic hypoxia is expected to increase in marine ecosystems, our experimental data suggest that cardiac performance may influence fish survival and distribution.

## 1. Introduction

Oxygen availability is a key driver of animal physiology, behaviour and ecology. Low levels of environmental oxygen (hypoxia) are a growing concern, especially in coastal waters, where the duration, frequency and severity of hypoxic events have increased in recent years [1,2]. Reduced oxygen availability negatively impacts fish, affecting their growth [3], embryonic development [4], sex ratio [5], predator–prey relationships [6], swimming activity [7] and niche utilization [8]. The European sea bass (*Dicentrarchus labrax*) is known to exhibit pronounced intraspecific variation in hypoxia tolerance which directly relates to survivorship in semi-natural tidal ponds [9]. This suggests that hypoxia tolerance is a determinant of individual fitness in sea bass. However, the factors underpinning intraspecific variation in hypoxia tolerance in this animal remain unclear.

Previous studies have drawn associations between measures of cardiac performance and organismal performance traits in fish [10,11]. For example, the cardiac power output of rainbow trout with exceptional swimming performance is significantly higher than that of poor swimmers [12]. Moreover, cardiac oxygen supply in many fish species, including sea bass, is precarious because they do not have a coronary blood supply but instead rely on the residual oxygen in returning venous blood [13]. Thus, cardiac hypoxia tolerance and whole-animal hypoxia tolerance should be strongly correlated in fish; although this hypothesis has not been directly tested.

Whole-heart contraction and relaxation is dependent on the cycling of Ca^2+^ in the muscle cells (cardiomyocytes) that constitute the heart. Ca^2+^ cycling is an energy-consuming process and hypoxia is known to depress contractility due to reduced respiration and adenosine triphosphate (ATP) turnover [14]. It is therefore possible that differences in myocardial Ca^2+^ handling underlie intraspecific variation in cardiac hypoxia tolerance. Here we test the hypothesis that individual variation in cardiac hypoxia tolerance is associated with individual variation in whole-animal hypoxia tolerance. Our second hypothesis is that superior heart performance during hypoxia is supported mechanistically through enhanced cellular Ca^2+^ handling, which we investigated pharmacologically.

## 2. Material and methods

Thirty-four European sea bass (*Dicentrarchus labrax*) (mass 286 ± 12 g, male) were obtained from a commercial supplier (Aquastream, Ploemeur, France) and maintained at *Institut Français de Recherche pour l'Exploitation de la Mer* (Ifremer) in Brest, France, where they were implanted with passive integrated transponders (PIT) to identify individual fish (see [9] for details).

Individual hypoxia tolerance was assessed using a hypoxia challenge test (HCT) which has been described in detail [9]. Briefly, nitrogen was used to decrease oxygenation from 100 to 20% air saturation over 90 min and by approximately 2% per hour thereafter. Once fish lost equilibrium, they were identified via PIT tag, and removed to recover in a fully aerated tank. The time taken to lose equilibrium and the corresponding oxygenation level was recorded. HCTs took place on 25th May 2012 (HCT1), 7th June 2012 (HCT2), 15th January 2013 (HCT3) and 12th December 2013 (HCT4). Directly following HCT4, the five most hypoxia-tolerant fish and the five most hypoxia-sensitive fish were used for the functional cardiac analyses.

Fish were killed with a blow to the head in accordance with local animal care protocols. The heart was removed, transferred to physiological saline and four myocardial muscle strip preparations were dissected from the ventricle with a razor blade. Each strip was hung between two vertical clips; the uppermost was attached to a force transducer, while the other provided a stable anchor. The muscle preparations were lowered into individual organ baths containing physiological saline maintained at 12°C, and stimulated to contract (10 ms, 70–85 V, 0.2 Hz). Two of the muscle strips were oxygenated and designated ‘normoxic’ and two were aerated to implement hypoxia. Our hypoxic conditions were designed to mimic the fivefold decrease in blood oxygen known to occur in sea bass exposed to 24 h of hypoxia [15]. See [16], and electronic supplementary material and methods for full experimental details.

All muscle preparations underwent three consecutive force–frequency trials. The first force–frequency trial was conducted in the presence of 1 nM adrenaline [17] and served as the control condition. In the second force frequency trial, one normoxic and one hypoxic preparation were treated with agents (10 µM ryanodine and 2 µM thapsigargin, [16]) that inhibit intracellular Ca^2+^ cycling through the sarcoplasmic reticulum (SR). The other muscle preparations served as time- and oxygenation-matched controls. For the third force frequency trial, all muscle preparations were exposed to a high dose of adrenaline (1 µM; [18]) which increases extracellular Ca^2+^ influx across the cell membrane. This drug treatment protocol was designed to test our second hypothesis and establish the role of intracellular Ca^2+^ (SR) and extracellular Ca^2+^ cycling in myocardial contractility during hypoxia.

HCT repeatability was assessed with the Pearson product-moment correlation coefficient. The myocardial force and kinetics were calculated as previously described [16]. Linear mixed-effects models (GLM) were used to analyse the relationship between whole-animal hypoxia tolerance and myocardial force of contraction, with force as the fixed factor, heart ID as a random factor and stimulation frequency as a categorical variable. Tukey's post-hoc test was used to determine significance (*p* < 0.05). All mean values are ±s.e.m.

## 3. Results

**Figure 1.**
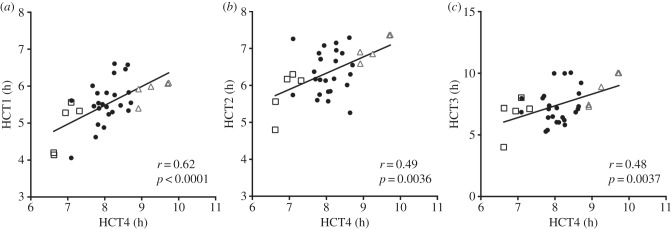
Individual whole-animal hypoxia tolerance is a stable trait in sea bass. Black squares (*n* = 5) represent fish subsequently designated HCT hypoxia-sensitive fish and grey triangles (*n* = 5) represent fish designated as HCT hypoxia-tolerant fish for myocardial preparation experiments. *R*-values and significance determined by Pearson's correlation coefficient (*N* = 34). HCT1 (May 2012); HCT2 (June 2012), HCT3 (January 2013) and HCT4 (December 2013).

Figure 1 shows individual hypoxia tolerance for each fish in the last HCT (HCT4) plotted against individual hypoxia tolerance in the previous three tests (HCT1–3). Individual hypoxia tolerance was temporally stable for the duration of the study (567 days) with certain fish consistently withstanding hypoxia longer (the ‘hypoxia-tolerant’ fish) and certain fish consistently succumbing to hypoxia earlier (the ‘hypoxia-sensitive fish’). HCT hypoxia-tolerant fish withstood HCT4 for greater than or equal to 8.9 h, whereas all HCT hypoxia-sensitive fish lost equilibrium within less than or equal to 7.5 h. The incipient lethal oxygen saturation for HCT hypoxia-tolerant fish was less than or equal to 3.2% air saturation, while it was greater than or equal to 3.8% air saturation for HCT hypoxia-sensitive fish.

**Figure 2.**
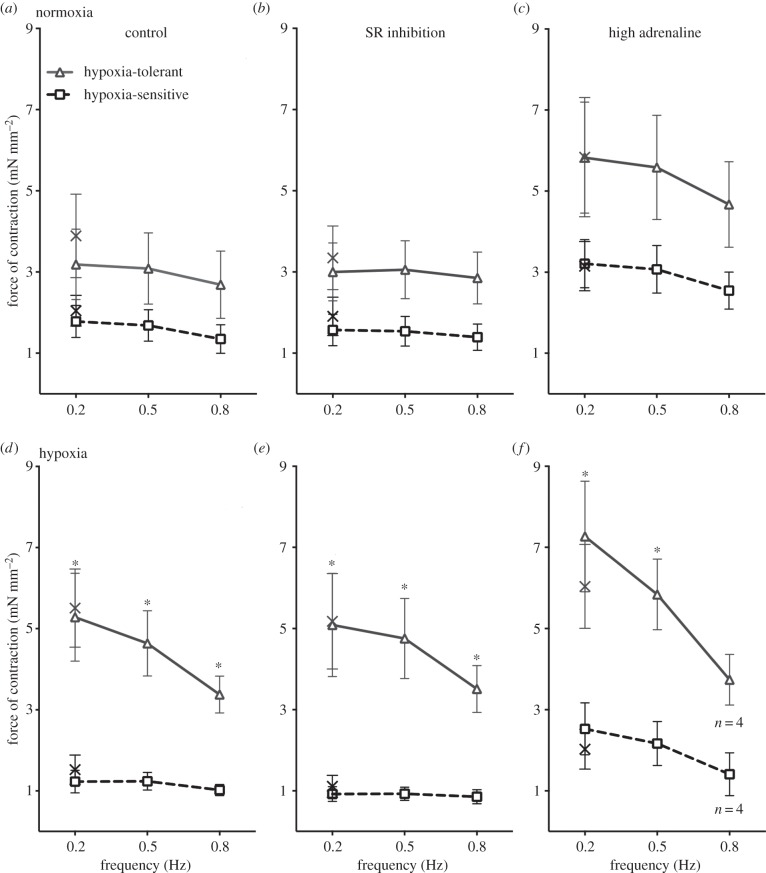
Myocardial muscle from hypoxia-tolerant fish outperforms that from hypoxia-sensitive fish under acute hypoxic exposure**.** Figure shows contractile force of cardiac muscle preparations from HCT hypoxia-tolerant fish and hypoxia-sensitive fish under normoxic conditions (*a*–*c*) and hypoxic conditions (*d*–*f*). (*a,d*) Myocardial force across physiologically relevant contraction frequencies under control conditions. (*b,e*) The effect of inhibiting intracellular Ca^2+^ cycling. (*c,f*) The effect of increasing extracellular Ca^2+^ influx with adrenaline (1 µM). The crosses show contractile force contraction upon return to 0.2 Hz after the frequency challenges. Values are means ± s.e.m. of *n* = 5 preparations. Asterisks denote difference between hypoxia-tolerant and hypoxia-sensitive fish (GLM).

In normoxia, we found no significant difference in the force produced by muscle preparations from HCT hypoxia-tolerant fish and hypoxia-sensitive fish under control conditions (figure 2*a*), after SR inhibition (figure 2*b*) or in the presence of high adrenergic stimulation (figure 2*c*). Likewise, rates of contraction or relaxation did not differ between HCT hypoxia-tolerant and hypoxia-sensitive fish at any frequency or with drug treatments under normoxia (table 1). However, under hypoxia, cardiac muscle preparations from hypoxia-tolerant fish produced significantly more force than hypoxia-sensitive fish under all conditions (control, figure 2*d*; after SR inhibition, figure 2*e*; and in the presence of high adrenaline, figure 2*f*). The difference occurred across all contraction frequencies (0.2, 0.5 and 0.8 Hz) but was most striking at the slow frequencies which are expected to occur *in vivo* during hypoxic bradycardia [19]. The stronger myocardial contractions under hypoxia in the HCT hypoxia-tolerant fish were accompanied by faster rates of contraction and relaxation than the HCT hypoxia-sensitive fish (table 1).

**Table 1.** Contractile kinetics under normoxia and hypoxia in ventricular muscle preparations from European sea bass fish in a given condition. Control saline contained 1 nM adrenaline, SR inhibition was achieved with 10 µM ryanodine and 2 µM thapsigargin and high adrenaline contained 1 µM adrenaline. In total, 0.8 Hz is representative of the *in vivo* heart rate of sea bass at 12°C and bradycardia (0.2 Hz) occurs during hypoxia. *n* = 5 in all cases except where marked with a dagger, in which *n* = 4. Bold typeface and asterisks denote a significant difference between hypoxia-tolerant fish and hypoxia-sensitive fish in a given condition (GLM).

	control				SR inhibition				high adrenaline			
	hypoxia-tolerant		hypoxia-sensitive		hypoxia-tolerant		hypoxia-sensitive		hypoxia-tolerant		hypoxia-sensitive fish	
normoxia	0.2 Hz	0.8 Hz	0.2 Hz	0.8 Hz	0.2 Hz	0.8 Hz	0.2 Hz	0.8 Hz	0.2 Hz	0.8 Hz	0.2 Hz	0.8 Hz
rate of contraction (mN mm^−2^ s^−1^)	5.71	6.43	3.21	3.23	5.74	7.48	2.83	3.42	10.17	11.88	5.38	5.84
	(±1.39)	(±1.78)	(±0.64)	(±0.80)	(±1.21)	(±1.48)	(±0.70)	(±0.74)	(±2.18)	(±2.50)	(±1.00)	(±1.01)
rate of relaxation (mN mm^−2^ s^−1^)	6.35	6.63	3.49	3.08	6.31	7.34	3.05	3.15	11.37	10.30	6.47	5.85
	(±1.72)	(±1.99)	(±0.60)	(±0.83)	(±1.53)	(±1.56)	(±0.66)	(±0.68)	(±2.70)	(±2.40)	(±1.03)	(±0.98)
resting tension (mN mm^−2^)	2.45	2.37	1.05	1.02	2.23	2.28	0.79	0.79	2.24	2.27	0.63	0.64
	(±0.85)	(±0.83)	(±0.49)	(±0.47)	(±0.84)	(±0.85)	(±0.38)	(±0.36)	(±0.85)	(±0.86)	(±0.36)	(±0.36)
hypoxia	0.2 Hz	0.8 Hz	0.2 Hz	0.8 Hz	0.2 Hz	0.8 Hz	0.2 Hz	0.8 Hz	0.2 Hz	0.8 Hz^†^	0.2 Hz	0.8 Hz^†^
rate of contraction (mN mm^−2^ s^−1^)	8.59	7.78	**2.16**	**2.68**	8.89	8.97	**1.71**	**2.15**	12.19	9.24	**4.22**	3.38
	(±1.76)	(±1.08)	(±0.47)*	(±0.34)*	(±2.14)	(±1.57)	(±0.33)*	(±0.42)*	(±2.29)	(±1.48)	(±0.97)*	(±1.05)
rate of relaxation (mN mm^−2^ s^−1^)	10.54	7.98	**2.77**	**2.71**	10.24	14.88	**1.59**	**2.09**	14.88	8.57	**5.49**	3.44
	(±1.85)	(±0.95)	(±0.64)*	(±0.51)*	(±2.49)	(±2.54)	(±0.34)*	(±0.40)*	(±2.54)	(±1.42)	(±1.44)*	(±1.35)
resting tension (mN mm^−2^)	1.74	1.77	0.37	0.38	1.41	1.44	0.29	0.35	1.31	1.39	0.34	0.06
	(±0.88)	(±0.90)	(±0.36)	(±0.37)	(±0.83)	(±0.82)	(±0.40)	(±0.41)	(±0.79)	(±1.08)	(±0.37)	(±0.39)

## 4. Discussion

Inter-individual variation in hypoxia tolerance remained stable for the duration of our study (567 days) indicating that acute hypoxia tolerance is a stable trait and a potential target for natural selection [20]. Indeed, fish mass increased fourfold between HCT1 and HCT4, indicating profound growth and phenotypic remodelling, while inter-individual hypoxia tolerance remained consistent. Hypoxia tolerance is a determinant of sea bass ecological performance under field conditions [9] and has been shown to have a genetic basis in other fish species (e.g. Atlantic salmon, [10]). We have shown separately [21] that robust performance under one environmental challenge test does not necessarily correlate with success in another (thermal tolerance and swimming capacity; and also see [9]). Moreover, there was no correlation between HCT performance and swimming performance in a preliminary study conducted on this cohort of sea bass (data not shown). In other words, although not directly tested here, the hypoxia-tolerant fish were not just better ‘all-rounders' than the hypoxia-sensitive fish. Although still to be identified, functional trade-offs with other performance traits are likely, which helps explain why both hypoxia-tolerant and hypoxia-sensitive fish persist over time in a population.

Myocardium from hypoxia-tolerant sea bass performed better under hypoxia than myocardium from hypoxia-sensitive sea bass, linking individual variability in hypoxia tolerance with individual variability in intrinsic cardiac performance. We reasoned that superior contractility under hypoxia would be due to greater involvement of the intracellular Ca^2+^ stores of the SR because the SR has been implicated in augmenting Ca^2+^ delivery to the heart during periods of stress [22]. However, we found no support for this hypothesis as inhibiting the SR did not affect force production or contractile kinetics under normoxia or hypoxia. Without Ca^2+^ cycling through the SR, the sea bass heart would depend wholly on extracellular Ca^2+^ for contraction. We show that increasing transmembrane Ca^2+^ flux with adrenergic stimulation (1 µM) increased force production, but did not explain the difference in individual performance during hypoxia.

Because we assessed myocardial performance *in vitro*, the mechanism underlying superior contractile performance of the hypoxia-tolerant fish in hypoxia must be intrinsic to the heart (i.e. not involving autonomic control). It is possible that variation in cardiac metabolism or myoglobin content may be related to hypoxic performance as fish with a high cardiac myoglobin concentration perform better during hypoxia [10,23].

This study suggests variation in cardiac hypoxia tolerance could, at least in part, underlie variation in whole-animal hypoxia tolerance. Although this finding is based on a small sample size, when it is considered in combination with the finding that hypoxia-tolerant fish have higher survivorship in semi-natural conditions [9], we suggest that the heart may be an important target for natural selection in a future where marine environments are oxygen-stressed. Indeed, we speculate that similar to growing evidence for temperature-tolerance [11], hypoxia tolerance of the fish cardiovascular system may be important in determining fish distribution and survival in the changing oceans.

## Supplementary Material

Supplemental Method

## Data accessibility

Raw data have been deposited in Dryad.
