# Supplementary material for: Individual variation in whole-animal hypoxia tolerance is associated with cardiac hypoxia tolerance in a marine teleost
Source: Biol Lett. 2016 Jan;12(1):20150708. doi: 10.1098/rsbl.2015.0708 (PMC4785915; doi:10.1098/rsbl.2015.0708)
Supplement: Supplemental Method [file rsbl20150708supp1.docx]

**Supplemental Materials and methods**

*Experimental Animals*

Thirty-four European sea bass (*Dicentrarchus labrax*) (mass 286 ± 12 g, determined as male after death) were obtained from a commercial supplier (Aquastream, Ploemeur, France) and maintained under natural photoperiod at *Institut Français de Recherche pour l’Exploitation de la Mer* (Ifremer) in Brest, France, in bio-filtered and thermoregulated (12 °C) sea water. Fish were fed daily with commercial feed (Le Gouessant, Lambale, France). A few weeks after their arrival, each fish was anaesthetized (2-phenoxyethanol; dilution 0.3 mL L^-1^) and a passive integrated transponder (PIT) tag was inserted subcutaneously to identify individual fish.

*Hypoxia Tolerance*

Individual hypoxia tolerance was assessed using a hypoxia challenge test (HCT) which has been described in detail elsewhere (see [[1](#_ENREF_1)]. Briefly, water in the holding tank was made hypoxic by bubbling with nitrogen so that oxygenation was rapidly (within 90 min) decreased from 100 % to 20 % air saturation and then gradually decreased further by approximately 2 % per hour. Once fish lost their ability to maintain balance/equilibrium, they were rapidly removed from the holding tank, identified via the PIT tag and allowed to recover in a fully aerated recovery tank. The time taken to lose equilibrium and the corresponding oxygenation level (the incipient lethal oxygen saturation) was recorded. HCTs took place on the following days: 25^th^ May 2012 (HCT1), 7^th^ June 2012 (HCT2), 15^th^ January 2013 (HCT3) and 12^th^ December 2013 (HCT4). Myocardial muscle performance experiments took place in December 2013.

Following HCT4, the 5 most hypoxia-tolerant ‘winner’ fish and the 5 least hypoxia-tolerant ‘loser’ fish were used for the functional cardiac analyses. HCT winners withstood HCT4 for ≥ 8.9 hours, whereas all HCT losers lost balance within ≤ 7.5 hours. Correspondingly, the incipient lethal oxygen saturation for HCT winners was ≤ 3.2 % air saturation, whilst it was ≥ 3.8 % air saturation for HCT losers.

*Myocardial Muscle Performance*

Sea bass were killed with a blow to the head followed by destruction of the brain in accordance with local animal care protocols. The heart was removed and transferred to ice-cold relaxing solution of the following composition (numbers in brackets are mM concentrations): NaCl (100); KCl (10); MgSO_4_ (4); KH_2_PO_4_ (12); taurine (50); glucose (20); and HEPES (10), adjusted to a pH of 6.9 with KOH. The ventricle was separated from the rest of the heart and weighed, before four myocardial muscle strip preparations were dissected clean with a razor blade.

Each muscle preparation was hung between two vertical clips; the uppermost was attached to a 25 g force transducer, whilst the other provided a stable anchor. Output from the force transducers was amplified (Transbridge 4M; World Precision Instruments) and converted to a continuously acquired digital signal using DataTrax data acquisition software (World Precision Instruments). The four muscle preparations were lowered into individual organ baths containing physiological solution (numbers in brackets are mM concentrations): NaCl (150); CaCl_2_ (2.0); KCl (5.4); MgSO_4_ (1.5); NaH_2_PO_4_ (0.4); glucose (10); and HEPES (10); adjusted to a pH of 7.7 with NaOH. Organ baths were maintained at 12 °C to match the holding temperature of the fish. After 20 minutes, the muscle preparations were stimulated (10 ms, 70-85 V) to contract at 0.2 Hz (12 bpm). Once the force of contraction had stabilized (30-90 minutes) the muscle preparations were progressively stretched until the maximum force of contraction was achieved (L_max_). Thus myocardial muscle contractility was assessed under peak isometric conditions.

Four ventricular muscle preparations from each animal were run in parallel, as is conventional for such studies [[2-4](#_ENREF_2)]. Two of these were oxygenated and designated ‘normoxic’ and two were aerated to implement hypoxia. There was a 5-fold difference in oxygen partial pressure between the normoxic (~84 kPa) and hypoxic baths (~20 kPa) and although these absolute values are higher than those found in the blood of sea bass, high levels are required *in vitro* due to the low oxygen carrying capacity of saline solution compared with blood [[5](#_ENREF_5)]. Importantly, the 5-fold difference between oxygen partial pressure in our normoxic and hypoxic groups is proportional to the 5-fold drop in arterial blood oxygen partial pressure in sea bass exposed to 24 hours of hypoxia [[6](#_ENREF_6)].

All muscle preparations underwent three consecutive force-frequency trials, where contractile performance was assessed at progressively faster pacing rates (0.2, 0.5, and 0.8 Hz). Force-frequency trials test the ability of the muscle to generate force at different rates and are an important intrinsic measure of myocardial performance [[7](#_ENREF_7)]. Frequencies of 0.5 and 0.8 Hz were chosen as they bracket the *in vivo* range of sea bass heart rate at 12 °C [[8](#_ENREF_8)], and 0.2 Hz is appropriate as fish heart rate slows during hypoxia (hypoxic bradycardia) [[9](#_ENREF_9)]. The first force frequency trial was conducted in the presence of 1 nM adrenaline, which mimics adrenergic tone in the resting circulation of fish [[10](#_ENREF_10)] and served as the control condition for this study. Subsequent force-frequency trials were conducted in the presence of drugs known to alter cellular Ca^2+^ cycling to investigate probable mechanisms underlying myocardial hypoxia-tolerance. In the second force frequency trial, one normoxic and one hypoxic muscle preparation were treated with agents that inhibit intracellular Ca^2+^ cycling through the SR. We used ryanodine at 10 µM to inhibit the SR Ca^2+^ release channel (ryanodine receptor) and thapsigargin at 2 µM to inhibit the Sarco(endo)plasmic Reticulum ATPase (SERCA2a) [[11](#_ENREF_11)]. The other muscle preparations served as time- and oxygenation-matched controls. For the third force frequency trial, all muscle preparations were exposed to a high but physiologically relevant dose of adrenaline (1 µM; [[12](#_ENREF_12)] which increases extracellular Ca^2+^ influx across the cell membrane [[13](#_ENREF_13)]. This drug treatment protocol was designed to test our second hypothesis and establish the role of intracellular Ca^2+^ (SR) and extracellular Ca^2+^ cycling in myocardial contractility during hypoxia.

1. Claireaux G., Theron M., Prineau M., Dussauze M., Merlin F.-X., Le Floch S. 2013 Effects of oil exposure and dispersant use upon environmental adaptation performance and fitness in the European sea bass, Dicentrarchus labrax. *Aquatic Toxicology* **130**, 160-170. (doi:10.1016/j.aquatox.2013.01.004).

2. Tiitu V., Vornanen M. 2002 Regulation of cardiac contractility in a cold stenothermal fish, the burbot Lota lota L. *Journal of Experimental Biology* **205**(11), 1597-1606.

3. Shiels H.A., Farrell A.P. 1997 The effect of temperature and adrenaline on the relative importance of the sarcoplasmic reticulum in contributing Ca2+ to force development in isolated ventricular trabeculae from rainbow trout. *Journal of Experimental Biology* **200**(#11), 1607-1621.

4. Imbert-Auvray N., Mercier C., Huet V., Bois P. 2013 Sarcoplasmic reticulum: a key factor in cardiac contractility of sea bass Dicentrarchus labrax and common sole Solea solea during thermal acclimations. *Journal of Comparative Physiology B-Biochemical Systemic and Environmental Physiology* **183**(4), 477-489. (doi:10.1007/s00360-012-0733-0).

5. Altimiras J.M., H; Gesser, H. . 1998 A quantitative analysis of oxygen diffusion limitations in myocardial tissue of the rainbow trout. . *Conference Proceedings The International Congress on the Biology of Fish* (Towson University, Baltimore (Maryland, USA)).

6. Thomas S., Hughes G.M. 1982 Effects of hypoxia on blood-gas and acid-base parameters of sea bass. *Journal of Applied Physiology* **53**(6), 1336-1341.

7. Bowditch H. 1871 Uber die Eigenthu¨ mlichkeiten der Reizbarkeit, welche die Muskelfasern des Herzens zeigen. Vorgelegt v.d.w. Mitgliede C. Ludwig. Berichte ber die Verhandlungen der ko niglich-sa¨chsischen Ge-sellschaft derWissenschaften zu Leipzig. *Mathematisch-physische Classe* **24**, 653-689,.

8. Axelsson M., Altimiras J., Claireaux G. 2002 Post-prandial blood flow to the gastrointestinal tract is not compromised during hypoxia in the sea bass Dicentrarchus labrax. *Journal of Experimental Biology* **205**(18), 2891-2896.

9. Farrell A.P. 2007 Tribute to P. L. Lutz: a message from the heart--why hypoxic bradycardia in fishes? *J Exp Biol* **210**(Pt 10), 1715-1725. (doi:210/10/1715 [pii]

10.1242/jeb.02781).

10. Graham M.S., Farrell A.P. 1989 The effect of temperature-acclimation and adrenaline on the performance of a perfused trout heart. *Physiological Zoology* **62**(#1), 38-61.

11. Shiels H.A., Vornanen M., Farrell A.P. 2002 The force-frequency relationship in fish hearts - a review. *Comparative Biochemistry and Physiology a-Molecular and Integrative Physiology* **132**(4), 811-826. (doi:10.1016/s1095-6433(02)00050-8).

12. Madzeau M.M., Madzeau F. 1982 Adrenergic responses to stress. In *Strss in Fish* (pp. 49-70. London, London Academic Press.

13. Shiels H.A., Vornanen M., Farrell A.P. 2003 Acute temperature change modulates the response of I Ca to adrenergic stimulation in fish cardiomyocytes. *Physiological and Biochemical Zoology* **76** (6), 816-824.
